# A Document Analysis of Peak Carbon Emissions and Carbon Neutrality Policies Based on a PMC Index Model in China

**DOI:** 10.3390/ijerph19159312

**Published:** 2022-07-29

**Authors:** Chenrui Lu, Bing Wang, Tinggui Chen, Jianjun Yang

**Affiliations:** 1 School of Statistics and Mathematics, Zhejiang Gongshang University, Hangzhou 310018, China; bulabula1127@163.com (C.L.); ctgsimon@mail.zjgsu.edu.cn (T.C.); 2 School of Artificial Intelligence and Electronic Commerce, Hangzhou College of Commerce, Zhejiang Gongshang University, Hangzhou 311500, China; 3 Department of Computer Science and Information Systems, University of North Georgia, Oakwood, GA 30566, USA; jianjun.yang@ung.edu

**Keywords:** peak carbon emissions, carbon neutrality, PMC, policy evaluation

## Abstract

With the commitment to peaking carbon emissions by 2030 and achieving carbon neutrality by 2060, the text analysis of policies related to peak carbon emissions and carbon neutrality has become a hot research topic in China. However, current domestic and foreign research mainly focuses on the impact and enlightenment of carbon emission measurement and other aspects and rarely optimizes the road to carbon neutrality through the analysis of policy texts. Based on both domestic and international research results, this paper takes 11 central government, ministry, province, and city policies as the research object, uses the PMC index model to calculate the PMC indices of the 11 representative documents, and draws surfaces. The results indicate that nearly half of the policies have excellent scores, but some policies still have deficiencies in terms of guarantee incentives and policy coverage. Given these shortcomings, this paper proposes that the government should provide technical assistance to industrial enterprises in addition to certain subsidies to reduce the energy consumption of enterprises in production and achieve sustainable development. While popularizing and developing low-carbon technology, enterprises should pay attention to personnel training and management, and use the digital economy to empower the transition to eco-friendly production. Based on the above research, the main contributions of this paper are as follows: to make theoretical and practical preparations for carbon neutralization and to provide suggestions for optimizing policies.

## 1. Introduction

Since the Industrial Revolution, due to the uncontrolled mining of coal and other traditional energy sources, ecological problems pose a severe threat to the development of all mankind. The emission of large amounts of greenhouse gases has led to global warming, causing a series of problems such as melting glaciers and rising sea levels. The Intergovernmental Panel on Climate Change believes that global warming must be controlled within 1.5 °C to curb this ecological disaster. Therefore, achieving carbon neutrality is a task of great urgency [1]. At present, more than 60 countries in the world are striving to achieve zero carbon emissions by 2050, and China’s goal of achieving Peak Carbon Emissions and Carbon Neutrality (PCECN) is a related initiative. At the general debate of the 75th Session of the United Nations General Assembly on 22 September 2020, President Xi Jinping announced that China would scale up its Nationally Determined Contributions (NDCs) by adopting more vigorous policies and measures, striving to peak CO_2_ emissions by 2030 and achieve carbon neutrality by 2060.

In this background, the analysis of PCECN policies has become a popular topic. Although many scholars analyzed the impacts of PCECN policies on enterprises, few analyzed the policy documents. In fact, by analyzing policy documents, we can mine the worthwhile content for the content characteristics of each policy. Therefore, policy document analysis plays a significant role in policy evaluation research.

Based on the PMC index (Policy Modeling Consistency index) model, this paper quantitatively evaluates and analyzes PCECN policies. This article has three-fold research significance. Firstly, based on a large number of policies, the comprehensive evaluation of the PCECN policies can better assist the government fulfilling the planning requirements for peaking carbon emissions by 2030 and achieving carbon neutrality by 2060 from both theoretical and practical perspectives. Secondly, the evaluation model of this research can analyze the pros and cons of the PCECN policies, thus improving the formulation and implementation of the corresponding policies. Thirdly, the policy-oriented research plays a pivotal role for enterprises in making their plans regarding energy conservation and emission reduction.

The structure of the paper is as follows: Section 2 is the literature review. Section 3 introduces the research framework of this paper. Section 4 uses the LDA (latent Dirichlet allocation) algorithm to extract the topics of public concern, selects indicators for evaluating the PCECN policy, and evaluates the effects of 11 PCECN policies based on the PMC model. Section 5 summarizes the full text and puts forward prospects for future work.

## 2. Literature Review

At present, the research on PCECN policies mainly focuses on four aspects: (1) the classification and discussion of PCECN policies; (2) the quantitative analysis method of policy documents; (3) different policy document analysis models in policy evaluation research; and (4) the relationships between enterprises and policies.

### 2.1. The Classification and Discussion of PCECN Policies

Different policy instruments can be classified according to different criteria. According to the evolution of environmental policy, Tietenberg [2] classified environmental policy instruments into three categories: command–control, market–incentive, and social–will. Luo [3] divided low-carbon policy instruments into three categories: economic incentive policy instruments, regulatory policy instruments, and social based on their role, evaluated the implementation effects of different policy instruments and their impacts on industrial development, and analyzed the frequency of various policy instruments. Generally speaking, a good policy often requires a comprehensive and balanced application of economic incentive policy instruments, regulatory policy instruments, and social policy instruments. However, China currently uses a large number of regulatory policy instruments, most of which rely on government coercive means and are short of flexible market regulation. The number of economic incentive policies is limited, i.e., functions of policy-related incentives, subsidies, and simulative resource allocation are less significant. Social policy instruments also have defects, resulting in insufficient government guidance and coordination and low social operation efficiency. For example, Yao [4] analyzed and studied 148 urban mineral-related policies from three perspectives: regulatory policy, economic incentive policy, and social policy. The study found that the various low-carbon policy instruments with their specific objects and conditions have advantages and disadvantages. In fact, to maximize the benefits of low-carbon policy instruments, the government needs to combine the characteristics of various low-carbon policy instruments and optimize the combination to build a sound low-carbon policy system. At the same time, for green development under the background of PCECN, enterprises need to strengthen the matching of policy instruments and communicate with policy departments, implement overall energy conservation and emission reduction, actively guide the diversified development of enterprises, and establish and improve enterprises’ transition and exit mechanisms under the new situation. It can be seen that the driving force and mechanism of policy instruments have also attracted the attention of many scholars.

### 2.2. The Quantitative Analysis Method of Policy Documents

There are four common methods for the quantitative analysis of policy documents: (1) the expert scoring method: Xu [5] used the expert scoring method to evaluate the policy logic consistency, economic sufficiency, political legitimacy, and implementation feasibility of industrial water pollution prevention and control policies in Wuzhou. (2) The questionnaire survey method: Douenne et al. [6] aimed to assess the prospects for French climate policies after the Yellow Vests crisis halted the planned increase in the carbon tax. Building on a new survey conducted on a sample of 3002 representative respondents of the French population, they elicited knowledge, perceptions, and values related to climate change, and they examined opinions on carbon taxation. (3) Web crawler technology: in order to explore the status of the Chinese public’s attention, changing trends, sentiment orientation, and focus toward green buildings, Liu et al. [7] collected and analyzed information from popular Weibo posts and comments related to green buildings. They used the Sina Weibo platform with web crawler technology and text mining. (4) Machine learning combining supervised and unsupervised algorithms: Li et al. [8] used SVM, random forest, plain Bayes, and other machine learning algorithms to train text to identify the emotional tendencies of new text and then judged sentiment polarity through unsupervised emotion analysis, with the aim of exploring the current status and temporal and spatial trends in public attention and sentiment orientation with a focus on recycled water in China. At present, there are many kinds of policy document analysis methods. When analyzing the same document, it is necessary to combine multiple document analysis methods organically to build a more systematic and effective policy document analysis framework.

In the past two years, with the increasing popularity of PCECN policies, domestic and foreign scholars have used various methods to evaluate PCECN policies. Wei et al. [9] comprehensively collated and investigated 1105 published research studies regarding PCECN through bibliometric analysis. From the space–time perspective, the authors quantitatively analyze the shifts in popular research trends. Chen et al. [10] took China’s pilot natural experiment as an example to test the policy effectiveness of carbon trading in two stages. The first stage employs the global DEA indicators to construct the total-factor carbon performance index and the energy-carbon performance index. The second stage uses the synthetic control method to discuss the effectiveness of carbon trading. Research shows that carbon trading can significantly improve performance. Xu et al. [11] collected panel data related to carbon emissions in 284 cities in China from 2003 to 2019, based on the difference-in-difference(DID) framework, and evaluated the energy-saving and emission-reduction(ESER) policies through data such as the logarithm of carbon emissions per capita. The research confirms that the carbon reduction effect of ESER policies could be achieved by promoting energy saving, optimizing structure, and strengthening green technology, which also reflects China’s efforts to deploy PCECN policy.

### 2.3. Research on Different Policy Text Analysis Models in Policy Evaluation Research

At present, domestic and international scholars use different models for text analysis. Cheng [12] collected and classified 143 environmental protection policies issued from 1982 to 2009; summed up six public values in the field of environmental protection policies in China; and carried out word segmentation, classification, and word frequency statistics for policy documents. Based on word frequency statistics, trend line fitting, factor analysis, and comprehensive scoring were used to analyze the policy’s value latitude and time dimension. Wang [13] used word frequency analysis to search for and analyze the carbon sink policy in Guangdong and evaluated the implementation intensity of carbon sink policy from target matching, realization path, operability, and clarity according to the annual average coefficient. Through the text analysis method, a policy text evaluation model is established based on the analytic hierarchy process, and the implementation effect of the carbon sink policy is evaluated through the policy score. Zhong [14] took 594 environmental policies as the research sample pool; adopted the theory of sustainable development, synergy theory, and the game theory of interests in environmental protection; and analyzed the effects of policy implementation from the annual changes in the release number, release frequency, and release departments. Following studying the release objects of environmental policy, policy implementation, policy evaluation, policy supervision, and policy instruments, the author put forward suggestions for the development of enterprises under a low-carbon background. Li [15] analyzed the current loopholes in China’s low-carbon policies from basic policy instruments and low-carbon competitiveness and proposed suggestions for efficient policy implementation and the future development of low-carbon policies.

In recent years, the PMC index model in various academic fields has become an effective method for evaluating policies. For example, Yang et al. [16] used the PMC model to quantitatively evaluate 11 policies that govern the new-energy vehicle industry in China and analyzed the impacts of the policies on the promotion of new-energy vehicles from the aspects of technological innovation policies, energy environmental policies, and tax subsidy policies. Dai et al. [17] used the PMC model to evaluate the effectiveness of the policy system for the green development of the Yangtze River Economic Belt. The research found that the green development policy system of the Yangtze River Economic Belt was relatively mature and played a fundamental role in a specific period. The relevant policies are of high quality in terms of completeness, rationality, and scientific foundations.

Based on text mining and deep learning methods, Zhao [18] selected 10 first-level variables: policy nature, guarantee incentive, policy field, policy function, coverage, public participation, function level, policy timeliness, communication and cooperation, and policy publicity to construct a PMC index model. The evaluation criteria for the energy policy portfolio are established to quantitatively evaluate the emission reduction policies. The conclusion is made that choosing a policy combining carbon trade and the carbon tax is the best response to carbon emission reduction. These studies show that compared with traditional methods such as AHP (analytic hierarchy process), the PMC model is more pertinent and operable, avoids the subjectivity of the evaluation process to a certain extent, and increases the accuracy of the quantitative evaluation of PCECN policies. Therefore, this paper uses the PMC model to provide a theoretical reference for PCECN policy optimization and innovation.

### 2.4. The Study of the Relationship between Enterprises and Policies

Some scholars study PCECN policies by analyzing the relationships between enterprises and policies. Hashim et al. [19] aimed to explore the roles of green process innovation and orientation toward environmental performance in achieving the long-term goal of carbon neutrality. In addition, their paper also discovered a mediating role of green competitive advantage in said context. The study employed structural equation modeling and found that green process innovation, environmental orientation and green competitive advantage significantly influenced environmental performance. Based on representative firm-level data for three countries, Austria, Germany, and Switzerland, Tobias et al. [20] investigated the effects of energy-related regulations, taxes, voluntary agreements, and subsidies on the creation of green energy products and analyzed through which channels policy affected green product innovation and which factors mediated the observed effects. Policy might affect green product innovation by directly stimulating the supply of green products/services or more indirectly by stimulating the demand for green products/services. Based on the background of coal industry development, Sun [21] analyzed the direction and possibility of coal industry development from the past to the future according to the SWOT principle.

Based on the above analysis, this paper uses the LDA topic model to extract the public’s concerns regarding the PCECN policies at first and then builds a PMC index model to score and evaluate some PCECN policies. Subsequently, the comprehensive text analysis method is adopted to evaluate China’s current PCECN policies. Finally, suggestions for improvement are made as well in this paper.

## 3. Research Framework

**Figure 1:**
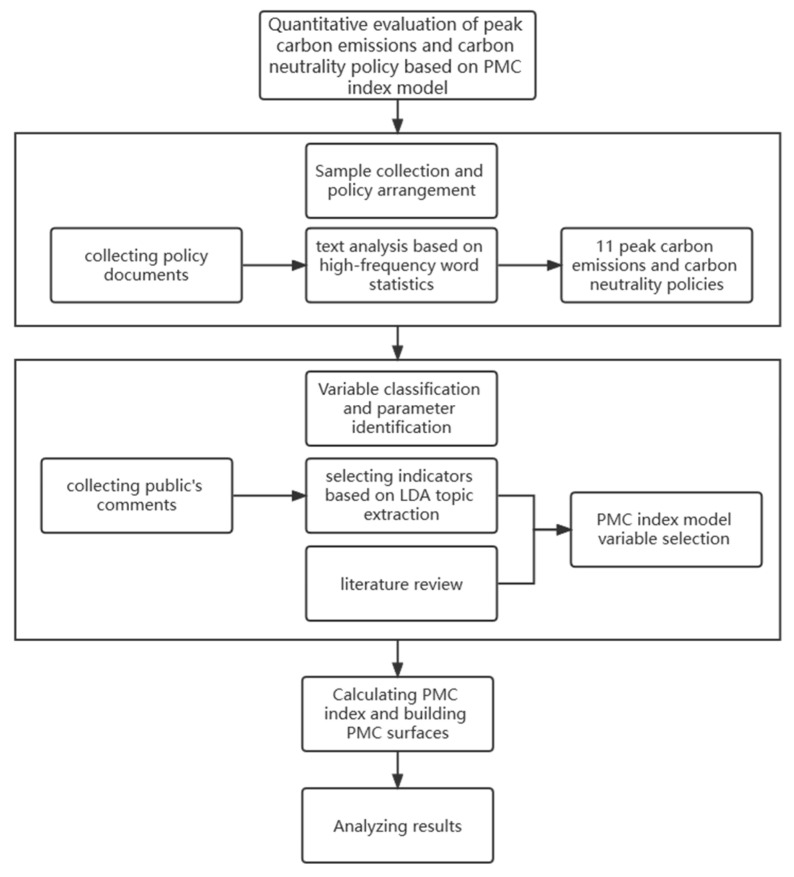
The research framework.

This paper first collects the PCECN policy documents from 2020 through web crawlers; then, it establishes the PMC index model, obtains its multi-input-output table based on 11 representative policy documents related to PCECN, and draws the PMC surfaces. Based on the PMC surfaces, the pros and cons of these 11 policies are analyzed, and countermeasures and suggestions are put forward for the development of carbon-peak and carbon-neutral policies. The research framework is shown in Figure 1.

## 4. Quantitative Evaluation of Policy Based on PMC Index Model

### 4.1. Sample Collection and Policy Arrangement

**Figure 2:**
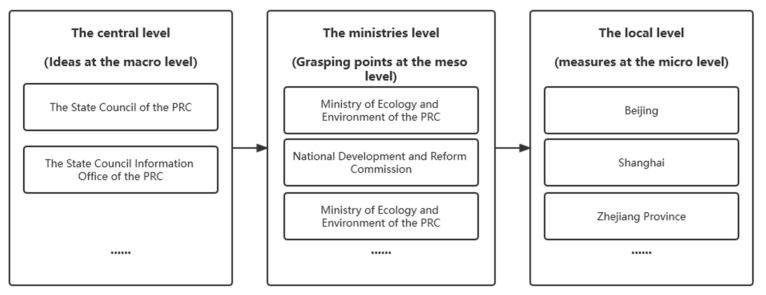
The roles and relationships of these main departments, ministries, commissions, and others.

Based on the authoritativeness principle, the PCECN policies in this paper are all collected from public official websites, including the General Office of the State Council, State Council Information Office of the People’s Republic of China, National Development and Reform Commission, and Ministry of Ecology and Environment of the People’s Republic of China and government portals. The policy documents include the PCECN policies issued by national ministries and local governments from September 2020 to March 2022. Through searching national and regional policy documents and for keywords related to carbon peak and carbon neutralization, 35 policies with related content were collected from President Xi Jinping’s speech and the General Office of the State Council; 13 were from the Ministry of Ecology and Environment and the National Development and Reform Commission; and 67 were from local governments. The roles and relations of these main departments, ministries, commissions, and others are shown in Figure 2. Among these 115 policy documents, policy documents at the central, ministry, and local levels are selected that represent the development goals of the government at all levels and demonstrate a guiding ideology for the carbon neutrality development of various industries. Specifically, five representative national policy documents, three ministry and commission policy documents, and three provincial and municipal policy documents were selected, covering ecological protection, traditional industrial transformation, carbon cycle economy, and other development themes. In terms of local policy selection, Beijing, Shanghai, and Zhejiang are selected because Beijing is the political center of China, Shanghai is the economic center of China, and Zhejiang is top-notch in ecological environment management and protection.

After that, this article converts these policies into plain text format for text preprocessing such as removing punctuation marks and then uses Python to perform word segmentation and high-frequency word statistics on the text. Finally, 11 PCECN policies including the Guiding Opinions on Accelerating the Establishment and Improvement of a Green, Low-Carbon and Circular Development Economic System are selected as research objects, as shown in Table 1.

### 4.2. Variable Selection Based on LDA Topic Extraction

Public opinion is significant when evaluating the merits of PCECN policy. Based on the LDA topic model, this section extracts the public’s comments on carbon neutralization and carbon peak videos on the Bilibili video website and the scrolling opinions in the bullet screens to determine the indicators for the policy quantitative evaluation model.

Bilibili is a well-known video comment website in China, with more than 200 million monthly active users by the end of 2021. Every user can post videos, share ideas, and express opinions on Bilibili, which is an important platform for studying the public’s attention to low carbon policy. This paper screened out videos related to the keyword “peak carbon emissions and carbon neutralization”, and based on the number of clicks, likes, bullet comments, and favorites, four videos were selected as the research objects and are shown in Table 2.

**Figure 3:**
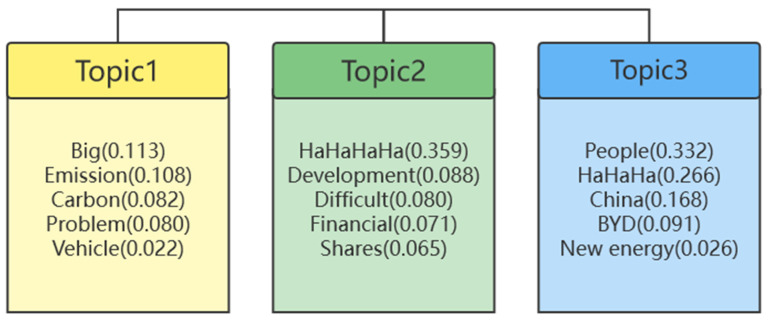
The topics and corresponding feature words.

Comprehensively sorting the comments and bullet screens of the four videos resulted in a total of 15,668 texts. Our implementation uses Python to clean the data and remove duplicate text data, expressions, special characters, and other redundant data. This finally resulted in10,520 valid data. When applying topic mining, Ida model of gensim in the Python package is adopted. The Python pseudocode of the process of LDA topic extraction is shown in Table 3. When the number of topics is set as three, each topic displays five words, and the probability of obtaining the topic and corresponding feature words is shown in Figure 3.

The mining results indicate that there are three potential topics for the comments on the four high-volume videos concerning peak carbon emissions and carbon neutralization: (1) carbon emission issues, (2) the financial and economic development in the PCECN, and (3) China’s new energy and electric vehicle BYD, briefly, green finance, carbon emission, and new energy. the mined topics based on the LDA model have high-precision keywords and strong independence, and it is easy to obtain related topics from the keywords.

### 4.3. Research Framework of PMC Index Model

The PMC index (Policy Modeling Consistency index) is based on the Omnia Mobilis hypothesis that everything in the world is interrelated; therefore, the selected indicators of the policy research model should include as many relevant variables as possible [22]. The PMC index model has two main features: first, it can analyze the internal consistency level of a policy; second, it can intuitively show the pros and cons of the policy and reflect the overall evaluation of the policy and the specific details of each item through the surface chart [22]. China’s PCECN policies are evaluated based on the PMC index model. The analysis steps are as follows [23]:

(1) Fill in the multi-input-output table with the primary variables and the secondary variables. There are 11 primary variables in this model, and each primary variable contains *n* secondary variables.
(2) Calculate each secondary variable that obeys the 0–1 Bernoulli distribution. When a keyword with a high word frequency in PCECN policy text is a secondary variable, the corresponding value is 1; otherwise, it is 0.
(3) Calculate the primary variable of the PMC model according to formula (1). The value of the primary variable is the average of the corresponding secondary variable values, and the calculated result is still in the range [0, 1]:

(1) $${\;X}_{i}\left(\overset{n}{\underset{j=1}{\sum }}\frac{X_{\mathrm{ij}}}{T\left(X_{\mathrm{ij}}\right)}\right),i=1,2,\ldots ,11$$

(4) Sum the values of all primary variables in the PMC model to obtain the PMC index of the policy.

### 4.4. Variable Classification and Parameter Identification

This paper takes the 11 screened policies related to PCECN as the research objects and quantitatively analyzes the rationality of each PCECN policy based on the PMC model. According to the research theory of Mario Arturo Ruiz Estrada, the PMC index is composed of primary variables and secondary variables. Because it is necessary to select as many primary variables and secondary variables as possible, the weight of each secondary variable in the PMC index model is equal. The topic extraction analysis demonstrates that the public pays more attention to carbon emission, green finance, and new energy. Therefore, variables can be introduced regarding these three items in establishing a policy evaluation model. The PMC index established here for the PCECN policies includes 11 primary variables, represented by X_1_~X_11_, as shown in Table 3.

In Table 4, the nature of the policy (X_1_) judges whether the PCECN policy can supervise, describe, aware, standardize, suggest, and pilot enterprises. Guarantee incentive (X_2_) judges whether the policy encourages enterprises or the public to participate in the implementation of the policy from the legal guarantee. Policy area (X_3_) examines the area in which the policy is implemented. Policy function (X_4_) judges whether the policy plays a functional role in environmental protection awareness. Coverage (X_5_) judges the policy participants. Application level (X_6_) judges the policy executor, whether it is the central government, provincial governments, or local governments. Release object (X_7_) examines which agency or department the policy is issued from. Policy portfolio (X_8_) examines whether the policy is implemented by multiple policy tools. Role level (X_9_) determines where the policy is affected. Policy timeliness (X_10_) examines the duration of policy implementation. Policy receptors (X_11_) refer to the beneficiaries of the policy, whether it is enterprises, governments, or residents.

### 4.5. Empirical Analysis of Quantitative Evaluation of PCECN Policy

Following the above process, the PMC indices and corresponding PMC surfaces of 11 peak carbon emissions and carbon neutrality policies can be acquired, and the PCECN policies can be evaluated according to the PMC indices. A score between 0 and 4.99 is bad, between 5 and 6.99 is good, between 7 and 8.99 is excellent, and between 9 and 11 is the best. Based on the existing policy text and the set parameters, the multi-input-output table of 11 policies is obtained, and the PMC indices of the policies are calculated and analyzed. Table 5 shows the calculation results of each dimension index.

**Figure 4:**
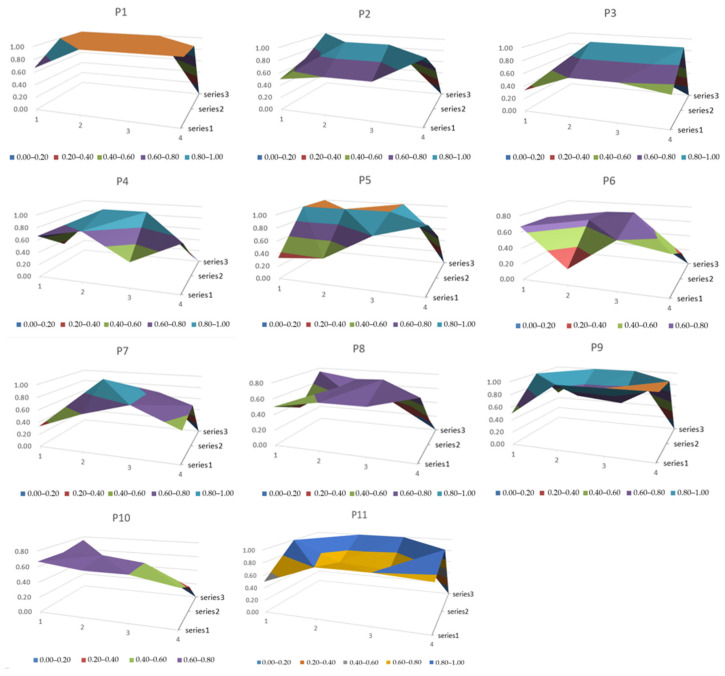
PMC surface of 11 PCECN policies.

Before constructing the PMC surface, the PMC matrix needs to be established. Since there are 11 variables, a 3 × 4 PMC matrix needs to be constructed, and the matrix value at the 12th position is set to 0. Figure 4 shows the PMC surfaces of the 11 PCECN policies.

As can be seen from Figure 4, these 11 policies are relatively reasonable, and their ratings are between best and good, reflecting that they play a positive role in contributing to the goal of peaking carbon emissions in 2030 and achieving carbon neutrality in 2060. Reducing pollution emissions and optimizing the energy consumption structure in various production fields through comprehensive policy combinations, the policies contribute to sustainable development. However, there are still some flaws in the existing policies. In particular, the current PCECN policies were launched in 2020, but their specific implementation plan still referred to the environmental management policies of the past 10 years. The average scores for policy coverage, policy portfolio, and guarantee incentives are lower than 0.7, and due to the lack of long-term planning, only 3 of the 11 policies have long-term validity. Therefore, to achieve long-term balanced development of PCECN in various fields, policymakers should keep improving the PCECN policies based on the deficiencies of existing policies and form policy systems that contribute to the healthy development of PCECN. The specific analysis is as follows:

Policies P1–P5 are all issued by the State Council and other state agencies. The PMC index forP1, Guiding Opinions on Accelerating the Establishment and Improvement of a Green, Low-Carbon and Circular Development Economic System is 10.67, and the best policy level, i.e., P1 considers guarantee incentives, policy areas, and policy functions comprehensively, guiding the establishment and improvement of green, low-carbon, and circular development in China. The PMC index of P3, Opinions on Deepening the Reform of Ecological Protection Compensation System, is the lowest because the policy is mainly aimed at the compensation system for the transition of high-energy-consuming enterprises to one of green development, which does not involve the development of new energy industry technology and has small policy coverage. The PMC indices for P2, Opinions on Establishing and Improving the Value Realization Mechanism of Ecological Products, and P4, Opinions on Promoting the Green Development of Urban and Rural Construction, are 8.37 and 7.12, respectively, both good; they focus on ecological products and urban and rural construction, respectively, resulting in a lower score in the field of policy application. The PMC index of P5, China’s Policies and Actions on Climate Change White Paper, is 8.53, an excellent rating. Since this document is a development white paper, its policy application fields are relatively wide, and it contains relevant regulations for multinational enterprises. At the functional level, it also encourages national innovation, regional economy, enterprise innovation, and the development of various industries.

Policies P6–P8 are related to PCECN and issued by the national ministries and commissions, which are related to ecological prevention and control of high energy consumption and high emission construction projects, energy consumption prevention and control, and industrial park carbon emission pilot policies. Consisting of control policies, they focus on carbon emission reduction and have lower requirements for the development of the new energy industry and low-carbon economy, so the rating is lower than the other 10 policies.

Policies P9–P11 are policies related to carbon neutralization in Beijing, Shanghai, and Zhejiang. The PMC surface shows that P10 has a low PMC index (6.05) because its policy functions and policy areas are focused on financial services. Therefore, policymakers can expand policy functions, refine financial services, take holistic steps to carbon neutrality in other fields, facilitate the financial industry’s carbon emission reduction support for traditional industries, and intensify the investment in new energy technologies under this policy. To expand the guarantee incentive effect of the policy, the government can also adopt various incentive measures to promote the development of financial services. Under P9, *Beijing’s 14th Five-Year Plan for National Economic and Social Development and Outline of 2035 Vision* (the outline of Beijing’s long-term goals), and P10, *Zhejiang Provincial Department of Ecology and Environment on Printing and Distributing Carbon Peak and Carbon Neutrality Work* (a medium- and long-term carbon neutrality development plan in Zhejiang Province), PMC indices such as guarantee incentives, policy areas, and policy functions are close to full marks, so the ratings are all high.

### 4.6. PMC Model Verification and Simulation Scenario Analysis

**Figure 5:**
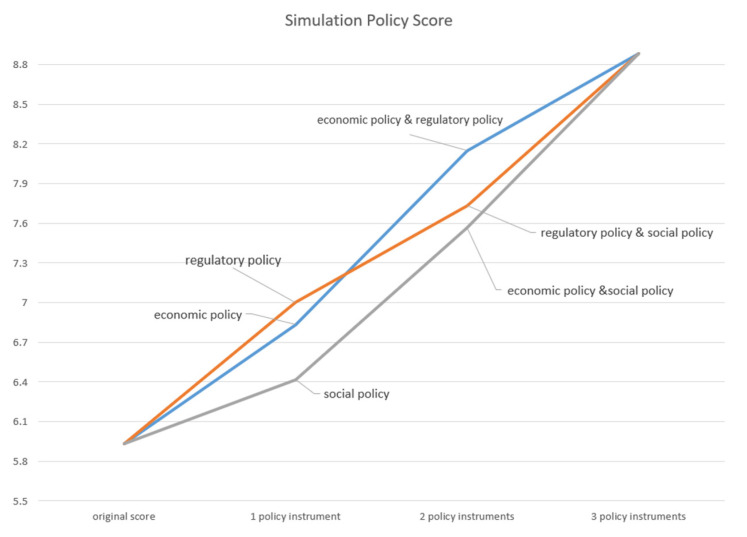
The impacts of different types of policy instruments on the P6 PMC index.

Based on the quantitative evaluation results for the PCECN policy texts, a PCECN policy scenario simulation model is constructed to examine the effectiveness of the model and analyze the effects of improving the policy. Since P6 is the policy with the lowest score, the simulation analysis is performed based on this policy. According to current research at home and abroad, the PCECN policies are mainly classified as economic incentive policy instruments, regulatory policy instruments, and social policy instruments. Therefore, the simulation experiment can be used to observe the change trend of the PMC index when the policy instruments are added. Table 6 shows the seven scenarios simulated by the simulation and the corresponding PMC index scores. Figure 5 shows the impacts of the different types of policy instruments on the P6 PMC index.

The construction of the PMC index model is to score the PCECN policy and analyze the advantages and disadvantages of the current policy so as to find the optimal development strategy and put forward targeted policy suggestions. It can be seen from Figure 4 that under the set parameter values, the simulation results of each combination policy are better than the original policy, which shows clearly that the policy regulation in these three aspects has promoted the development of PCECN to a certain extent. The change in the PMC index caused by the influence of the three types of policy instruments reflects the good validity of the PMC model.

At the same time, the simulation shows that for P6, when the three policy instrument types are combined at the same time, its PMC index is 8.88, and the policy level is excellent, which has a good role in promoting the development of PCECN. However, compared with the remaining 10 policy documents above, its ranking is fourth, affected by factors such as the policy timeliness and the role level of the policy. This shows that the PMC model has good stability and robustness.

## 5. Conclusions and Prospects

### 5.1. Conclusions

By constructing a PMC index model for policy analysis, this paper analyzes 11 representative policy documents related to PCECN and draws the corresponding PMC surfaces. It can be seen from the results that nearly half of the 11 policy documents have high ratings, while policies with only good ratings are defective in guarantee incentives and policy coverage. Therefore, the government can further optimize the existing PCECN policies. Based on the analysis results from the above PMC index model, the following conclusions can be drawn:

(1) According to the evaluation ratings obtained by the PMC index model, the selected 11 representative policy documents all score well or above. For policies with lower ratings, most of them score lower in guarantee incentives, policy coverage, etc. Therefore, while issuing policies, the government should strengthen the guarantee incentive mechanism and expand the policy functions.
(2) In response to the lack of social guidance in the policy field, the government should urge enterprises to reduce carbon emissions and scientifically advance the low-carbon transition of the energy industry. During the green transition of enterprises, the government should attach great importance to small and medium-sized enterprises in industry and construction. In addition to providing relevant preferential policies and incentive mechanisms, the government should provide technical support for small and medium-sized enterprises to help them reduce energy consumption and improve energy utilization and thus promote these SMEs to realize sustainable development.
(3) In response to the lack of economic guidance in the policy field, the government should inject vitality into green finance, and enterprises should give full play to the role of the market, refine internal control management, and make scientific decisions to contribute to the development of a green economy. The government can expand green credit, develop green insurance, and optimize a green financial system. While building a green internal cycle, the government can explore cross-border carbon trade financing and encourage the energy industry to reduce resource emissions.
(4) In response to the shortage of technical guidance in guarantee incentives, the government can advance the development of the new energy industry and encourage enterprises to innovate technology in energy conservation and emission reduction. At this time, the government should step up the popularization and independent research and development of new energy equipment, ensuring that the current new energy products meet market expectations and balancing the mismatching supply and demand. In addition, the government must cooperate with enterprises, attach importance to talent management, and cultivate core technological advantages to seize the technology market. New energy companies can also use digital technology to empower the low-carbon transition and explore business opportunities for low-carbon technologies.
(5) In response to policy coverage aiming at enterprises, to promote the publicity of environmental protection awareness in the public, the government should first popularize the concept of green consumption among consumers and enhance their awareness of environmental protection. At the same time, the government should intensify publicity regarding new energy, transform the public’s consumption, and expand the market for green consumption. In terms of policy assistance to multinational companies, China should facilitate exchanges and cooperation with other countries in green technology, green equipment, and green services; lower thresholds for multinational companies; and contribute to the export of green development experience.

### 5.2. Limitations and Prospect

In establishing the PMC index model, this paper quantitatively analyzes 11PCECN policies, summarizes the strengths and weaknesses of the PCECN policies based on the comprehensive evaluation results, and proposes optimization suggestions for the PCECN policies. However, there are some limitations in this study as follows:

(1) The data for text analysis come from laws and regulations issued by government departments. However, the government’s environmental policies can also be reflected in other aspects, such as news stories, conferences, and other media. As such, the data sources can be further enriched. Moreover, when this paper selected the national representative policy documents related to PCECN, it chose three local documents from Beijing, Shanghai, and Zhejiang provinces with good economic development and governance capabilities. Therefore, more comprehensive documents should also be collected for further study, especially from some poor economic regions.
(2) Since the PCECN has only been issued for two years, the number of current policies is relatively limited. However, with the further launch of PCECN, policy texts will have certain differences within a certain period, which can provide new ideas and perspectives for the study of policy texts.
(3) For the PMC model, the indicator setting still has certain room for improvement. To make the model more universal, nonstandard variables can be added, and quantitative analysis can be carried out according to the specific conditions of different policies; then the application scope of the PMC index model can be expanded [24]. The calculation method of the PMC index only calculates a simple summation and average without integrating the information between the policy evaluation indicators, so it cannot measure the relationships between different evaluation indicators [25].

The following can be explored in future research:

(1) Increase the number of PCECN policy documents in different regions, compare the similarities and differences of PCECN among regions, and provide more targeted policy optimization suggestions.
(2) Over time, the study can increase the comparative analysis of the time dimension [26].
(3) Establish a PMC index model by capturing the nonlinear relationships between the indicators [27] based on a neural network to make up for the defect of the same proportion between the indicators of the PMC model.

## Figures and Tables

**Table 1:** The PCECN policies for analysis.

Number	Release Time	Release Subject and Name
1	22 February 2021	The State Council of the PRC issued *Guiding Opinions on Accelerating the Establishment and Improvement of a Green, Low-Carbon and Circular Development Economic System*
2	26 April 2021	The State Council of the PRC issued *Opinions on Establishing and Improving the Value Realization Mechanism of Ecological Products*
3	12 September 2021	The State Council of the PRC issued *Opinions on Deepening the Reform of Ecological Protection Compensation System*
4	21 October 2021	The State Council of the PRC issued *Opinions on Promoting the Green Development of Urban and Rural Construction*
5	27 October 2021	The State Council Information Office of the PRC released *China’s Policies and Actions on Climate Change White Paper*
6	30 May 2021	Ministry of Ecology and Environment of the PRC issued *the guiding opinions on strengthening the prevention and control of the ecological environment source of high energy consumption and high emission construction projects*
7	11 September 2021	National Development and Reform Commission issued *the plan for improving the dual control of energy consumption intensity and total amount*
8	28 October 2021	Ministry of Ecology and Environment of the PRC issued *Notice on carrying out pilot carbon emission assessment in the environmental impact assessment of Industrial Park Planning*
9	13 March 2021	Beijing issued the *Beijing’s 14th Five-Year Plan for National Economic and Social Development and Outline of 2035 Vision*
10	8 October 2021	Shanghai issued *the opinions on accelerating the construction of Shanghai as an international green financial hub to serve the PCECN goals*
11	17 September 2021	Zhejiang Province issued *Zhejiang Provincial Department of Ecology and Environment on Printing and Distributing Carbon Peak and Carbon Neutrality Work*

**Table 2:** Research object video title and related data.

Title	Time	Clicks	Likes	Bullet Comments	Favorites	Forward
Carbon has not reached the peak, and people have reached the peak, see how the new energy industry can win under PCECN background	5 June 2021	388,729	25,416	8844	4340	2240
How will China’s carbon neutrality plan affect the lives and work of this generation?	4 May 2021	977,170	66,852	41,000	23,000	17,000
[carbon neutralization] from China to the world, Nature can’t sit still	9 April 2021	466,435	20,824	3796	7660	1861
Academician Ding Zhongli: Research on China’s “carbon neutrality” framework Roadmap	4 June 2021	416,567	31,174	12,000	14,000	5257

**Table 3:** The Python pseudocode of the process of LDA topic extraction.

import numpyas np from genism import corpora, models
if _name_= ’_main_’: input documents build dictionary calculate text vector calculate document TF-IDF LDA model fitting print topic distribution of documents print word distribution and its probability of three topics

**Table 4:** Variable classification of the PMC index model.

Primary Variables	Number	Secondary Variables	Primary Variables	Number	Secondary Variables
The nature of the policy	X_1_:1	Supervise	Application level	X_6_:1	central government
	X_1_:2	Describe		X_6_:2	provincial governments
	X_1_:3	Aware		X_6_:3	local governments
	X_1_:4	Standardize	Release object	X_7_:1	The State Council of the PRC
	X_1_:5	Suggest		X_7_:2	The State ministries and commissions of the PRC
	X_1_:6	Pilot enterprises		X_7_:3	Provincial and municipal prefectural committees
Guarantee incentive	X_2_:1	Legal protection		X_7_:4	Provincial and municipal departments and bureaus
	X_2_:2	Tax incentives		X_7_:5	Other
	X_2_:3	Technology guidance	Policy portfolio	X_8_:1	One
	X_2_:4	Investment subsidies		X_8_:2	Two
	X_2_:5	Talent support		X_8_:3	More than two
Policy area	X_3_:1	Economic	Role level	X_9_:1	National innovation
	X_3_:2	Social		X_9_:2	Regional economic
	X_3_:3	Technology guidance		X_9_:3	Industrial development
	X_3_:4	Environmental		X_9_:4	Enterprise innovation
	X_3_:5	Politics	Policy timeliness	X_10_:1	Long-term
Policy function	X_4_:1	Carbon emission reduction		X_10_:2	Medium-term
	X_4_:2	Technology innovation		X_10_:3	Short-term
	X_4_:3	Economic benefits	Policy receptors	X_11_:1	Enterprise
	X_4_:4	Environment awareness		X_11_:2	Government
Coverage	X_5_:1	Individual		X_11_:3	Residents
	X_5_:2	Domestic enterprises			
	X_5_:3	Multinational enterprises			

**Table 5:** PMC index model results.

Policy	P1	P2	P3	P4	P5	P6	P7	P8	P9	P10	P11	Average
The nature of the policy	0.67	0.50	0.33	0.67	0.33	0.67	0.33	0.50	0.50	0.67	0.50	0.52
Guarantee incentive	1.00	0.60	0.60	0.80	0.40	0.20	0.60	0.60	1.00	0.60	0.80	0.65
Policy area	1.00	0.60	0.60	0.40	0.80	0.60	0.80	0.60	1.00	0.60	0.80	0.71
Policy function	1.00	1.00	0.50	0.75	1.00	0.50	0.50	0.75	1.00	0.50	0.75	0.75
Coverage	1.00	0.67	0.33	0.33	1.00	0.67	0.33	0.33	1.00	0.67	1.00	0.67
Application level	1.00	1.00	1.00	1.00	1.00	0.67	1.00	0.67	0.67	0.67	0.67	0.85
Release object	1.00	1.00	1.00	1.00	1.00	0.80	0.80	0.80	0.60	0.60	0.60	0.84
Policy portfolio	1.00	0.67	1.00	0.33	0.67	0.33	0.67	0.33	1.00	0.33	1.00	0.67
Role level	1.00	1.00	0.50	0.50	1.00	0.50	0.50	0.75	0.50	0.75	0.75	0.70
Policy timeliness	1.00	0.67	0.67	0.67	0.33	0.67	0.67	0.67	1.00	0.33	1.00	0.70
Policy receptors	1.00	0.67	0.33	0.67	1.00	0.33	0.67	0.33	1.00	0.33	1.00	0.67
PMC index	10.67	8.37	6.87	7.12	8.53	5.93	6.87	6.33	9.27	6.05	8.87	7.72
Rank	1	5	8	6	4	11	7	9	2	10	3	
Policy level	The best	Excellent	Good	Excellent	Excellent	Good	Good	Good	The best	Good	Excellent	

**Table 6:** The simulation and corresponding PMC index scores.

Simulation	Economic Incentive Policy Instruments	Regulatory Policy Instruments	Social Policy Instruments	PMC Index
original policy	/	/	/	5.933333
Simulation 1	+	/	/	6.833333
Simulation 2	/	+	/	7
Simulation 3	/	/	+	6.416667
Simulation 4	+	+	/	8.15
Simulation 5	+	/	+	7.566667
Simulation 6	/	+	+	7.733333
Simulation 7	+	+	+	8.883333

## Data Availability

The data used to support the findings of this study are available from the corresponding author upon request.
